# Laminin Polymerization and Inherited Disease: Lessons From Genetics

**DOI:** 10.3389/fgene.2021.707087

**Published:** 2021-08-12

**Authors:** Liam Shaw, Conor J. Sugden, Kevin J. Hamill

**Affiliations:** Institute of Life Course and Medical Sciences, University of Liverpool, Liverpool, United Kingdom

**Keywords:** laminin, netrin, Pierson syndrome, MDC1A, basement membrane, junctional epidermolysis bullosa

## Abstract

The laminins (LM) are a family of basement membranes glycoproteins with essential structural roles in supporting epithelia, endothelia, nerves and muscle adhesion, and signaling roles in regulating cell migration, proliferation, stem cell maintenance and differentiation. Laminins are obligate heterotrimers comprised of α, β and γ chains that assemble intracellularly. However, extracellularly these heterotrimers then assemble into higher-order networks *via* interaction between their laminin N-terminal (LN) domains. *In vitro* protein studies have identified assembly kinetics and the structural motifs involved in binding of adjacent LN domains. The physiological importance of these interactions has been identified through the study of pathogenic point mutations in LN domains that lead to syndromic disorders presenting with phenotypes dependent on which laminin gene is mutated. Genotype-phenotype comparison between knockout and LN domain missense mutations of the same laminin allows inferences to be drawn about the roles of laminin network assembly in terms of tissue function. In this review, we will discuss these comparisons in terms of laminin disorders, and the therapeutic options that understanding these processes have allowed. We will also discuss recent findings of non-laminin mediators of laminin network assembly and their implications in terms of basement membrane structure and function.

## Introduction

Basement membranes (BMs) are flexible 40–120 nm sheets that separates cells from underlying connective tissue and regulate important cell behaviors such as cell polarity and migration, metabolism, and in inducing differentiation (Paulsson, 1992). Most BMs consist of two layers; an electron-lucent layer, lamina lucida comprised predominantly of laminins (LMs) and nidogens, and an electron dense layer, lamina densa of type IV collagen (col IV) and perlecan (Paulsson, 1992). BMs assemble through a multistep process, with the LM network assembling first (Kalb and Engel, 1991; Smyth et al., 1998; Li et al., 2002, 2005; McKee et al., 2007, 2009) *via* anchoring of the LMs cell surface receptors (Kalb and Engel, 1991; Smyth et al., 1998; Li et al., 2002, 2005; McKee et al., 2007, 2009). Anchorage increases local LM concentration, allows polymerization and recruitment of other components to the LM scaffold.

## The Laminins

Laminins are an obligatory feature of every BM. Each LM is an αβγ heterotrimer comprised of one of five α chains (encoded by LAMA1-5), one of four α chains (LAMB1-4) and one of three γ (LAMC1-3) chains (Aumailley et al., 2005; Domogatskaya et al., 2012; Aumailley, 2013). Use of alternative promoters in LAMA3 gives rise to either the short LMα3A or the longer LMα3B form, which are functionally and structurally distinct (Ryan et al., 1994; Ferrigno et al., 1997). Restrictions in heterotrimerization potential means that only 16 αβγchain combinations are possible (Paulsson et al., 1985; Engel et al., 1991; Nissinen et al., 1991; Utani et al., 1994). These are differentially expressed and play context specific roles. For example, α1β1γ1 (LM111) is essential for embryonic development and knockout of any of those genes is not compatible with life, whereas α3Aβ3γ2 (LM332) is highly expressed in mature epithelial tissues and loss of function leads to epidermal fragility.

**FIGURE 1:**
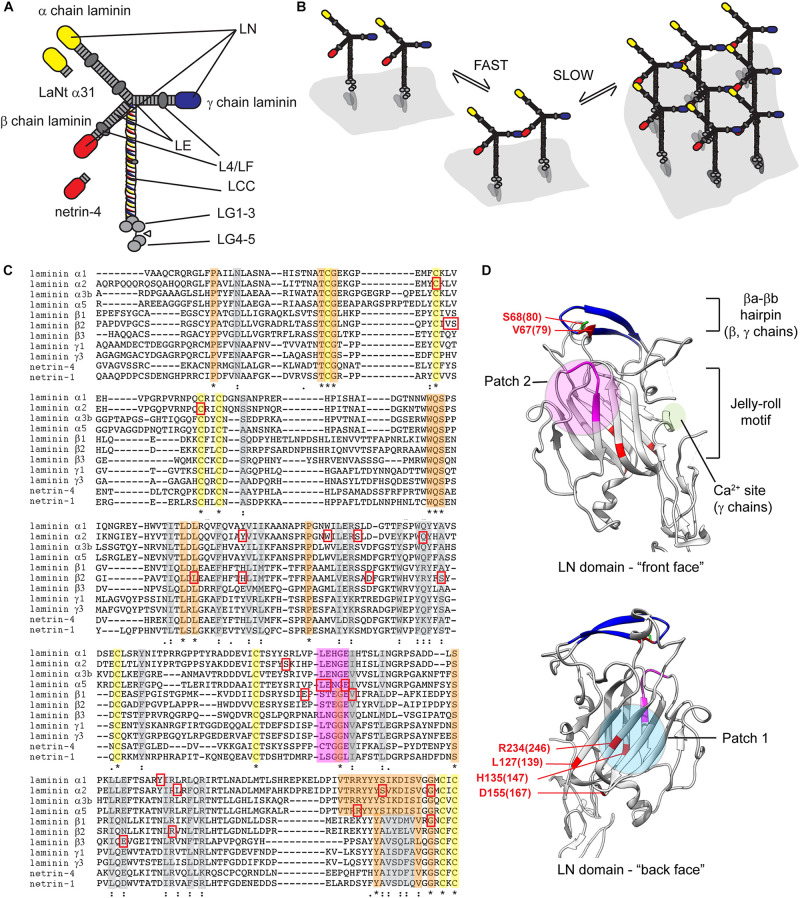
**(A)** Diagrammatic representation of archetypal LM structure. Yellow ovals αLN domains, red = βLN domains and blue = γLN domains. LE, laminin-type EGF-like repeat; LCC, laminin coiled coil domain; LG, laminin globular domains. **(B)** Two-step laminin network assembly. Secreted LMs associated with cell surface receptors, predominantly *via* their LG domain regions, β−γ LN domain interactions are then facilitated in a reaction with rapid on/off kinetics, then in the propagation step β−γ interactions are stabilized *via* incorporation of an α-chain leading to a stable cell-associated network. **(C)** Sequence alignments of LN domains from human laminin chains, netrin-1 and netrin-4. Yellow, orange, and gray highlights indicated conserved cysteines, fully conserved residues, or partially conserved residues. Magenta highlight identifies LENGE region. Red squares indicate pathogenic missense mutations. **(D)** Crystal structures of LN domains. View of the front and back face of the β1 chain is shown with features of conserved patches involved in LN-interaction (patch 2) and required for domain folding (patch 1). Amino acids associated Pierson syndrome mutations are indicated numbered based on LMβ1 with LMβ2 equivalent in parenthesis. Crystal structures derived from Hussain et al. (2011) and Carafoli et al. (2012) rendered using UCSF chimera (Pettersen et al., 2004). * = conserved residue, : = conservation of residues with strongly similar properties,. = conservation of residues with weakly similar properties.

The LM family has arisen by gene duplication and rearrangement, and common structural motifs are shared between members (Figure 1A). Archetypal LM chains consists of a laminin N-terminal domain (LN domain) followed by rod-like stretches of epidermal growth factor-like repeats (LE domains) interspersed with globular domains (L4 or LF domains) and followed by a coiled coil domain (LCC domain) through which αβγ heterotrimers form (Paulsson et al., 1985; Engel et al., 1991; Nissinen et al., 1991; Zimmerman and Blanco, 2007). In α chains, the LCC domain is followed by five LM globular domains (LG1-5), which harbor the highest affinity cell surface receptor sites (Timpl et al., 2000). While this architecture holds true for most LMs, not all chains contain all domains. Importantly, the α3A, α4, and γ2 chains contain shorter amino terminal arms lacking LN domain (Aumailley et al., 2005; Hamill et al., 2009).

### LN Domains

Laminin N-terminal domain are 252–264 amino acid globular domains found not only in LMs but also other ECM proteins including netrins. 14% of all residues in LN domains are conserved with strict conservation of six key cysteines. There is 72% homology between LMα1 and LMα2, 77% between LMα3B and LMα5, 72% between LMβ1 and LMβ2, and 64% between LMγ1 and LMγ3 LN domains. Lowest conservation is between LMβ3 and the β1 and β2 LN domains (38 and 42%, respectively) (Garbe et al., 2002).

Laminins interactions have been studied over many years with important early work establishing a “three-arm” model; polymerization only occurs when all the constituent chains contain LN domains (Yurchenco and Cheng, 1994; Hohenester and Yurchenco, 2013). Moreover, interactions must be heterotypic, involving an α, β and γ LN domain (McKee et al., 2007). The assembly process is divided into a temperature-dependent oligomerization step and a calcium-dependent polymerization step. *In vitro* analyses have further shown that the αβγ ternary node assembly involves rapid but unstable formation of βγ pairs that are then stabilized through integration of an αLN domain (Hussain et al., 2011; Figure 1B). In line with the three-arm model, LMs that lack one or more LN domain cannot polymerize independently (Hohenester and Yurchenco, 2013). These include LM332 and LM411, which are abundantly expressed in many epithelial and endothelial BMs. For these LMs, alternative methods of interaction with other LM isoforms may be required for BM assembly. For LM332, incorporation into skin BM can be partly explained by an interaction between LM332’s β3LN domain with the LE domain of α3 in LM311. These LM dimers could then self-associate into higher order networks (Champliaud et al., 1996; Rousselle and Beck, 2013). Non-network forming LM BM incorporation likely also depends on compensatory interactions with other BM/ECM components, such as the β3LN domain binding of the NC1 domain of type VII collagen, nidogen binding to the γ1 LE repeats (Chen et al., 1997; Rousselle et al., 1997; Rousselle and Beck, 2013).

The crystal structures of α5, β1, γ1 LN domains have been solved, and these combined with conservation of residues between chains allows inferences as to which regions are involved in domain folding and those involved αβγ ternary node formation (Figure 1C). The crystals revealed a similar overall structure of an antiparallel β sandwich with 8 β sheets forming a jelly roll motif held in conformation by cysteines C200 and C220 (Figure 1D; Hussain et al., 2011; Carafoli et al., 2012). In the α5LN domain, two conserved motifs Patch 1 and Patch 2 are of particular relevance (Hussain et al., 2011). Patch 1 within the conserved β1-β2-β7-β4-β5 “back face,” consists of E178, P189, R265, and R267. These residues are blocked by a glycan attached to N148 (Hussain et al., 2011), suggesting that Patch 1 plays a structural, non-polymerizing role (Carafoli et al., 2012). Patch 2 is located across the β6-β3-β8 “front face,” residues W132 and N168, and the β5-β6 loop, residues P229, L230, and E231. Patch 2 is not glycosylated nor conserved with β- or γ-chains but is important for polymerization as mutation of PLENGE residues in the β5-β6 loop all result in inhibition of polymerization (Hussain et al., 2011; Carafoli et al., 2012). The β-sandwiches of β1LN and γ1LN domains have similar structure with the main differences in peripheral regions (Carafoli et al., 2012). β1LN contains two particular regions of functional importance: the βa-βb hairpin and the β7-α4 loop. The βa-βb hairpin sits at the top of the domain with S80 a key residue for β-γLN interactions (Purvis and Hohenester, 2012). One notable difference in γ1LN domain is a calcium binding site located within a short α-helix and flanked by highly conserved D106 and T114 (Carafoli et al., 2012). Testing inferences about residue function is a laborious task but elegant *in vitro* analysis using LN domain- fusion proteins have been performed and improve interpretation of clinical findings, discussed in context below (McKee et al., 2018).

Laminin N-terminal domains, in addition to polymerization, are implicated in cell adhesion, neurite outgrowth, perlecan, heparin and heparan sulfate binding (Nielsen and Yamada, 2001; Nomizu et al., 2001; Garbe et al., 2002; Kunneken et al., 2004). The LN domain of LMα1, α2, and α5 can interact with integrins α1β1, α2β1, and α3β1, and presumed between LMα3b and integrin α3β1 based on antibody inhibition (Pfaff et al., 1994; Colognato et al., 1997; Ettner et al., 1998; Kariya et al., 2004; Hozumi et al., 2012). These interactions are lower affinity than LG domain interactions and likely are involved in localization to allow polymerization rather than signal propagation.

### Laminin LN Domains and Human Genetic Disease

The importance of LN domains to tissue function becomes apparent when the variety of LN domains mutations which lead to human disease are considered. Each affected LM results in a distinct set of syndromic disorders reflecting isoform-specific distribution and functions (Table 1).

**TABLE 1:** Pathogenic LN domain mutations.

Protein	Mutation	Effect	Phenotype	References
LMα1	Y265C	LN interaction	[mouse] Retinal vasculopathy	Edwards et al., 2010
LMα2	C79R	LM poly/fold^c^	[mouse] mild muscular dystrophy	Patton et al., 2008
	C86Y	fold^p^	MDC1A	Oliveira et al., 2018
	Y138H	LM poly^p^	MDC1A	Oliveira et al., 2008
	W152G	fold^p^	Limb-girdle–type dystrophy	Gavassini et al., 2011
	S157F	fold^p^	MDC1A	Geranmayeh et al., 2010
	Q167P	LM poly^c^	Limb-girdle–type dystrophy	Di Blasi et al., 2005
	S204F	fold^p^	Mild muscular dystrophy, mild proximal weakness	Chan et al., 2014
	L243P	fold^p^	Mild MDC1A	Gavassini et al., 2011
	S277L	fold^p^	MDC1A	Beytia Mde et al., 2014
	G284R	LM poly^p^	limb-girdle–type dystrophy	Gavassini et al., 2011
LMα5	R286L	LM poly^c^	Focal segmental glomerulosclerosis, hearing loss, craniofacial dysmorphism, limb development	Jones et al., 2020
LMβ1	E215K	LM poly^c^	[fly] heart development defects	Hollfelder et al., 2014
	V226E		[fly] heart development defects	Hollfelder et al., 2014
	G286R		[fly] heart development defects	Hollfelder et al., 2014
LMβ2	R246W R246Q	LM Secretion/Fold	End-stage renal disease, nephrotic proteinuria, diffuse mesangial sclerosis, focal and segmental glomerulosclerosis, microcoria, lens abnormalities, nystagmus hypotonia, cognitive defects, muscle delay	Zenker et al., 2004; Hasselbacher et al., 2006; Bredrup et al., 2008; Matejas et al., 2010
	V79del	LM poly^p^	Retinal detachment, cataracts, progressive vision loss, diffuse mesangial sclerosis, end-stage renal disease	Matejas et al., 2006
	S80R	LM poly^c^	Nephrotic proteinuria, atypical diffuse mesangial sclerosis, myopia, retinal detachment. [mouse S83R] Detrimental on Alport syndrome background	Matejas et al., 2010; Funk et al., 2018
	H147R	LM fold^p^	Nephrotic proteinuria, diffuse mesangial sclerosis, proliferative glomerulonephritis, hypertension, heart failure, microcoria, retinal detachment, lens abnormalities	Mohney et al., 2011
	D167Y	LM Secretion^p^	End-stage renal disease, myopia, retinal detachment, severe visual impairment	Kagan et al., 2008
	L139P	LM fold^p^	Diffuse mesangial sclerosis, lens abnormalities, severe visual impairment, hypotonia, muscle delay, cognitive deficits	Matejas et al., 2010
	S179F	LM fold^p^	End-stage renal disease, focal and segmented glomerulosclerosis, retinal detachment, severe visual impairments	Choi et al., 2008
LMβ3	E210K	Splicing^c^ + fold^p^	Skin fragility, nail dystrophy, alopecia	Mellerio et al., 1998

*^*p*^predicted ^*c*^confirmed *in vitro* assays.*

#### LAMB2 Mutations (Pierson Syndrome)

Pierson syndrome first described in 1963, is a severe congenital nephrotic syndrome with eye abnormalities (Pierson et al., 1963), caused by mutations in LAMB2 (LMβ2). LMβ2 is highly expressed in the glomerular basement membrane, multiple ocular structures (lens, retina, and cornea), and neuromuscular synapses (Hunter et al., 1989; Noakes et al., 1995; Libby et al., 2000; Bystrom et al., 2006). In addition to the defining features of congenital nephrotic syndrome that rapidly progresses to end-stage renal failure, and microcoria, many patients develop complications of motor delay, speech difficulties, intellectual disability, and seizures (Bowen et al., 1964). Indeed, the spectrum of LAMB2-related phenotypes is vast. The severest forms of the disease are associated with knockout mutations, whereas the majority of missense and indel mutations cluster to the LN domain and result in milder forms of the disease (Table 1).

One of the earliest studied LMβ2 LN mutations, R246W, is characterized by severe end-stage renal disease and microcoria (McKee et al., 2018). Similarly, R246Q, resulted in severe glomerular abnormalities and impaired secretion (Chen et al., 2011). Conservation of this arginine led to predictions that mutations impair LM polymerization, and *in vitro* this mutant polymerize substantially less effectively than the wild type protein (Zenker et al., 2004). However, R246W also reduced abundance of LM in BMs, likely due to disturbed protein processing (Matejas et al., 2010). Together these data indicate that this arginine has a role in protein folding. A second highly studied missense mutation, S80R, affects the highly conserved βa-βb and directly prevents LN–LN domain interactions with polymerization disrupted *in vitro* (Matejas et al., 2010; Carafoli et al., 2012; Purvis and Hohenester, 2012). Again, the importance of this region was further highlighted by an adjacent V79del patient (Matejas et al., 2006), presenting with milder symptoms of atypical diffuse mesangial sclerosis, retinal detachment, and myopia.

Other β2LN mutations with variable phenotypes include H147R (Mohney et al., 2011), L139P (Matejas et al., 2010), D167Y (Kagan et al., 2008), and S179F (Choi et al., 2008). Similar to R26Q, H147R caused a partial reduction in polymerization ability *in vitro*. L139P and D167Y mutations are near each other and are predicted to affect LN domain folding, and together suggest this region to be particularly sensitive to changes. L139P interferes with the hydrophobic core, is associated with a particularly severe phenotype. In contrast, the D167, and H147 result in milder phenotypes.

#### LAMA2 Mutations – (Merosin-Deficient Congenital Muscular Dystrophy Type 1A)

Mutations affecting α LN domains affect the stabilization step of ternary node assembly. The best example is merosin-deficient congenital muscular dystrophy type 1A (MDC1A), caused by mutations to LAMA2 (LMα2) (Helbling-Leclerc et al., 1995). This affects LM211 and LM221, the most abundant LMs in skeletal muscles (Ehrig et al., 1990), peripheral nerves, astrocytes and pericytes in the brain (Voit et al., 1995).

In LMα2 knockout conditions, MDC1A presents with disabilities of the proximal and distal limb muscles, with patients unable to walk more than a few steps unaided (Philpot et al., 1995; Jones et al., 2001). Weakness in facial muscles result in reduced sucking and swallowing capabilities, life-threatening problems can arise from failure of the respiratory muscles (Mendell et al., 2006), and cases with intellectual disability and epilepsy have been reported (Philpot et al., 1995; Muntoni and Voit, 2004; Mendell et al., 2006). In knockout situations, LM411 replaces LM211 in muscle basement membranes (Ringelmann et al., 1999). LMα4 lacks an LN domain and is unable to polymerize, resulting in a weakened BM. LMα4 and LMα2 also differ in their receptor binding interaction repertoire and affinities (Talts et al., 2000), for example, LMα2 binds integrin α7β1 whereas LMα4 cannot, and LMα4 has weaker affinity for α-dystroglycan (Nishiuchi et al., 2006). Comparison between missense mutations and knockout mutations allows differentiation between polymerization and receptor-mediated effects, although these inferences are complicated by not every affected tissue expressing LM411.

Many mutations have been reported throughout LAMA2’s 65 exons in MDC1A and are cataloged in LAMA2 gene variant database^1^ (Oliveira et al., 2018). Again, the LN domain contains a cluster of missense and in frame deletions (Patton et al., 2008; Oliveira et al., 2018). For example, a point mutation in the highly conserved CxxC motif, C79R, led to a milder form of MDC1A, which affects the myelination of Schwann cells in spinal roots and the stability of the skeletal muscles (Patton et al., 2008). This amyelination was not attributed to a change in abundance or mislocalization, and *in vitro* assays confirmed a dramatic effect on LM polymerization (McKee et al., 2018). Other pathogenic missense variants include Q167P, Y138H, G284R on the surface of α2 LN domain and C86Y, W152G, S157F, S277L, S204F, L243P in the interior (Yurchenco et al., 2018; Table 1). The S204F mutation lies at one extreme of the phenotypic spectrum, whereby the patient was misdiagnosed with a peripheral neuropathy, presenting with mild proximal weakness. Muscle biopsy revealed depletion of LMα2 in intramuscular nerve, subtly depleted LMα2 expression in muscle BMs and diffusely upregulated LMα5 expression (Chan et al., 2014). To the other extreme, Q167P maps to near the polymerization face, and consistent with this, causes a 60% drop in *in vitro* polymerization capability. This leads to ambulatory muscular dystrophy (McKee et al., 2018). More severe still, G284R causes proximal weakness, with a loss of functional gait with age accompanied by frequent falls, and epilepsy. The mutation effect is yet to be confirmed but predicted to inhibit LM polymerization (Gavassini et al., 2011).

#### LAMA5 Mutations (Kidney, Craniofacial, and Limb Development Syndrome)

LMα5 is almost ubiquitous to all adult BMs. Unsurprisingly, knockout mice die before birth with a failure in neural tube closure, and no human knockouts have been reported (Miner et al., 1998). However, a patient with R286L in LMα5 LN has been identified. They presented with a complex syndromic disease characterized by defects in kidney, craniofacial and limb development (Jones et al., 2020). The affected residue lies adjacent to the conserved PLENGE sequence required for LM polymerization (Hussain et al., 2011), and R286L abrogated *in vitro* polymerization potential (Jones et al., 2020). We cannot compare the LN mutation against knockout; however, a patient with V3140M, in the LG3 domain has been reported (Sampaolo et al., 2017). Both the LG3 mutation and R286L lead to complex syndromic disorders with similarity in tissues affected but with notable differences. Specifically, in the skin V3140M caused alopecia, lack of eyebrows and body hair, features not present in the R286L patient. V3140M patients also had retinal rod degeneration whereas the R286L had hearing loss but no sight abnormalities. Kidney defects were common to both with R286L presenting with atypical focal segmental glomerulosclerosis progressing to end stage kidney disease compared with floating kidney syndrome in V3140M. Finally, R286L presented with numerous dysmorphic issues include craniofacial dysmorphism, syndactyly, and pyloric web.

#### LAMB3 Mutation (Junctional Epidermolysis Bullosa)

LMβ3 is expressed in most epithelial tissues where it forms part of LM3a32 and LM3b32 (Matsui et al., 1995; Ferrigno et al., 1997). The resulting heterotrimers have either one or two LN domains and are unable to polymerize independently (Yurchenco and Cheng, 1994; Cheng et al., 1997). One would assume that LN domain mutations are tolerated for this LM chain. However, patients were identified where the pathogenic mutation caused E210K, which gives rise to a phenotype of trauma-induced blisters, nail dystrophy and alopecia (mild junctional epidermolysis bullosa) (McGrath et al., 1995; Mellerio et al., 1998; Posteraro et al., 2004). In comparison, homozygous knockout of LMβ3 leads to much more extensive skin blistering complications and early lethality (Pulkkinen et al., 1994; Kuster et al., 1997; Ryan et al., 1999; Meng et al., 2003).

Interpretation of the E210K mutation is complicated; the affected base pair is at a splice junction and in a knock-in mouse model led to skipping of the out-of-frame, and no detectable LMβ3 in the skin. However, in humans, miss-splicing has been reported for some, but not all patients, which can be rescued by second-site mutations (Pasmooij et al., 2007). Numerous alternative splice products are produced, including some full-length transcripts. Modeling of the E210K mutation indicates it is unlikely to be required for laminin polymerization but also is not predicted to affect protein folding or secretion (Mittwollen et al., 2020). The most common in-frame deletion is predicted to remove several of the central β-strands and disrupt the fold. Overall, the evidence from these patients does not point toward a LM polymerization effect but does suggest a role for the LMβ3 LN domain in protein function. Direct evidence for the importance of the LMβ3 LN domain has been obtained from keratinocytes expressing either full-length or LN domain-deleted LMβ3 and grafted as skin equivalents onto immunodeficient mice (Sakai et al., 2010). Here, the LN deleted versions displayed subepidermal blistering, erosions, and prominent granulation tissue, not associated with reduced LM332 deposition pointing LN domain roles beyond polymerization.

### Non-human LN Domain Mutations

LMα1 is extremely important for developmental processes, with knockout mice embryonic lethal. However, Y256C mice are viable with retinal defects of vitreal fibroplasia, vascular tortuosity and hypervascularization, and abnormalities to the retinal inner limiting membrane (Edwards et al., 2010). No reduction in LMα1 was noted, and a two-hybrid screen identified the mutation affects LN–LN interaction. Random mutagenesis in Drosophila has identified three LN domain mutations in LMβ1 that led to heart developmental defects, E215K, V226E, and G286R (Hollfelder et al., 2014). Of these, E215K was tested in *in vitro* assays and reduced polymerization (McKee et al., 2018).

### LM Network Regulators: Netrin-4 and LaNt α31

The netrins family of proteins are structurally and ancestrally related to LMs (Tessier-Lavigne and Goodman, 1996; Fahey and Degnan, 2012). Each netrin comprises a LN domain and stretch of LE repeats followed by a unique C-terminal region (Kappler et al., 2000; Yurchenco and Wadsworth, 2004). The LN domains of most netrins have diverged that they do not influence LM network assembly. However, for netrin-4 the situation is dramatically different where the β-type LN domain of netrin-4 can potently disrupt LM networks (Schneiders et al., 2007; Reuten et al., 2016, 2021). The physiological implications of this ability are beginning to be appreciated; recent work has demonstrated that netrin-4 levels are a key determinant of basement membrane stiffness with knock-on effects to cell behavior and tumor metastasis (Reuten et al., 2016, 2021).

Whereas netrins have evolved as independent genes, alternative splicing from LM genes or proteolytic processing of LM proteins leads to generation of LN domain containing fragments (Kariya et al., 2004; Hamill et al., 2009; Horejs et al., 2014). These fragments contain “perfect” LN domains that are likely to compete for binding sites (with reduced potency compared with netrin-4). One LAMA3-derived alternative splice isoform, Laminin N terminus α31 (LaNt α31) has widespread expression in human tissues (Troughton et al., 2020b), is upregulated during wounding and corneal limbal stem cell activation (Barrera et al., 2018) and emerging data indicate that it can modulate LM organization *in vitro* (Troughton et al., 2020a). *In vivo* overexpression is embryonic lethal during development with tissue defects that resemble LM network disruption phenotypes (Sugden et al., 2020).

From an evolutionary perspective, netrin-4, LaNt α31 and proteolytically released LN fragments represent multiple mechanisms to fine-tune LM network assembly. Although human diseases directly associated with loss-of-function mutations have not been identified, a SNP in the netrin-4 gene (causing Y205H) has been associated with late onset Alzheimer’s disease (Saad et al., 2015), and dysregulation of expression appear to contribute to tumor pathogenesis and point toward an additional important aspect of BM biology (Schneiders et al., 2007; Reuten et al., 2016, 2021; Troughton et al., 2020c).

### Rescuing LN Domain Defects

Although the standard gene and protein therapy toolbox are available to treat LN domain disorders, the large size of LM genes and associated challenges of producing and delivering recombinant therapy-grade LM protein presents challenges. However, promising results have been obtained recently from delivering the 800 kDa LM521 to the blood stream of LAMB2-null mice which rescues some aspects of Pierson syndrome. The delivered LM521 accumulated in the glomerular basement membrane in the correct orientation and led to reduced expression of the podocyte injury markers, and delayed the onset of proteinuria. However, the exogenous LM521 did not migrate to the podocytes nor fully restore the glomerular filtration barrier. Smaller, or hybrid proteins, may be a solution to overcome these challenges (Lin et al., 2018). For some LM disorders, upregulating expression of a compensatory LM may be a viable option. While there are differences, LMα1 and LMα2 are very similar both structurally and functionally, therefore in LMα2-deficient MDC1A, increasing LMα1 could compensate for the lack of functional LMα2. LMα1 expression is usually downregulated following development; however, encouraging progress has been made here using guide RNA to target the LMα1 promoter with inactive Cas9 coupled to VP160 transcription activation domain. In mouse models, electroporation of the gRNA-containing plasmids into the tibialis anterior of 4-week old animals led to increased expression of LM111 with appropriate localization 2-weeks post-electroporation (Perrin et al., 2017). This data provides an encouraging base for development that may be exploitable for other conditions using a similar approach.

A particularly innovative solution exploiting the knowledge gained from studying LM polymerization and counteracting the inherent LM size problems is using protein chimeras to act as linkers (McKee et al., 2009, 2017; Reinhard et al., 2017). Three such “Frankenstein” chimeric proteins have been created, a fusion of a functional LN domain to the LM binding region of nidogen, a miniature form of agrin (mini-agrin) containing only the LM-binding regions and α-dystroglycan binding regions, and a fusion between LM-binding domains of agrin and the dystroglycan binding domain of perlecan. As LM411 is upregulated in MDC1A but cannot compensate for LM211 dysfunction, the nidogen/LN domain chimeric protein can be used to bind the γ1 chain of LM411 *via* the nidogen region and provide the missing αLN domain needed to allow LM411 polymerization (Reinhard et al., 2017). The mini-agrin/perlecan chimeras can be used in concert with the nidogen chimera to compensate for α-dystroglycan binding (Talts et al., 2000; Moll et al., 2001). Where patients harbor LN mutations, only the nidogen fusion would be required, whereas for knockout both the LN/nidogen and mini-agrin would be necessary. Promising results have been observed with these chimeras in mouse models. Moreover, switching the LN domain from an αLN to βLN, this approach is likely to also be effective for Pierson syndrome patients.

## Discussion and Perspectives

Comparison between knockout and missense mutation associated phenotypes in LM genes has provided valuable information to identify which LMs are essential for individual tissues, but also which domains are involved. Rather than a binary outcome caused by ability or inability to polymerize, we see system-wide differences highlighting the multifaceted roles of LN domains. The variety of pathologies arising from mutations within a stretch of ∼250 amino acids illustrate the importance of LN domains to tissue function.

## Author Contributions

LS, CS, and KH wrote and edited the manuscript. All authors read and approved the final version of the manuscript for publication.

## Conflict of Interest

The authors declare that the research was conducted in the absence of any commercial or financial relationships that could be construed as a potential conflict of interest.

## Publisher’s Note

All claims expressed in this article are solely those of the authors and do not necessarily represent those of their affiliated organizations, or those of the publisher, the editors and the reviewers. Any product that may be evaluated in this article, or claim that may be made by its manufacturer, is not guaranteed or endorsed by the publisher.

## Footnotes

1
https://www.lovd.nl/LAMA2
